# The contribution of antinuclear antibodies in Primary Biliary Cholangitis (PBC): An experience from the immunology laboratory at University Hospital Center Hassan II, Fes, Morocco

**DOI:** 10.5339/qmj.2023.sqac.27

**Published:** 2023-11-26

**Authors:** I. El Mitri, H. Kaaouch, M. Ouboks, O. Ballil

**Affiliations:** ^1^Laboratory of Immunology, Central Laboratory Services; Hassan II Hospital Fes; Morocco Email: b.elmitri@gmail.com; ^2^Faculty of Medicine, Pharmacy, and Dentistry, Sidi Mohamed Ben Abdallah University, Fes, Morocco

## Abstract

Primary biliary cholangitis (PBC) is a chronic autoimmune cholestatic liver disease. If this disease is not treated, it might lead to fibrosis, cirrhosis, and its associated complications.^1^ Early recognition of this entity and immediate institution of therapy are the primary prognostic modifiers.^2^ Anti-nuclear autoantibodies can be used for diagnosis, particularly the anti-gp 210 and anti-Sp100 antibodies. Our aim in this study is to describe the epidemiology, clinical, biochemical, and immunological aspects of patients with PBC, thus, emphasizing the role of those antibodies.

**Methods:**

Patients older than 18 with chronic hepatitis or cirrhosis were recruited from September 2021 to January 2023. Clinical data were collected, including age, sex, symptoms, and biochemical parameters (ALT), (AST), (ALP), (GGT), (TB), (PT) and (PN), autoantibodies associated with hepatic autoimmune disorders: (AMA), anti-gp 210, anti-Sp100 and anti-F-actin antibodies were identified using HEp-2 cells as substrate (Fig. 1); and/or multiple Immunodot liver tests. HBsAg, anti-HCV, and HIV test results of the patients were found to be negative.

**Results:**

19 out of 71 had a confirmed diagnosis of PBC; 5 patients with AIH-PBC overlap (26.3%), and 14 patients with PBC (73.7%). Patients mainly were females with a 0.12-sex ratio. ANA patterns were dominated by cytoplasmic filamentous staining (AC-21) with punctuate nuclear envelope staining (AC-12). Multiple Immunodot tests were performed on all patients: AMA was present in 89.4%, anti-gp 210, and anti-Sp100 antibodies in 57.8% and 21.1%, respectively (Table 1).

Patients were divided into groups depending on the positivity of AMA, anti-gp 210, and anti-Sp100 antibodies. 10.5% of PBC patients (2) had negatives AMA and positive anti-gp210. Almost half of PBC patients (11) had associated positive AMA and positive anti-gp210 and/or anti-Sp100, while 26.3% of them (5) had only positive AMA (Table 2).

**Conclusion:**

The findings in this work support the role of anti-gp 210 and anti-Sp100 in identifying patients with PBC and overlap syndromes. Moreover, Anti-gp 210 antibodies are found to be helpful for the diagnosis of PBC patients who are negative for AMAs.

## Figures and Tables

**Figure 1.**
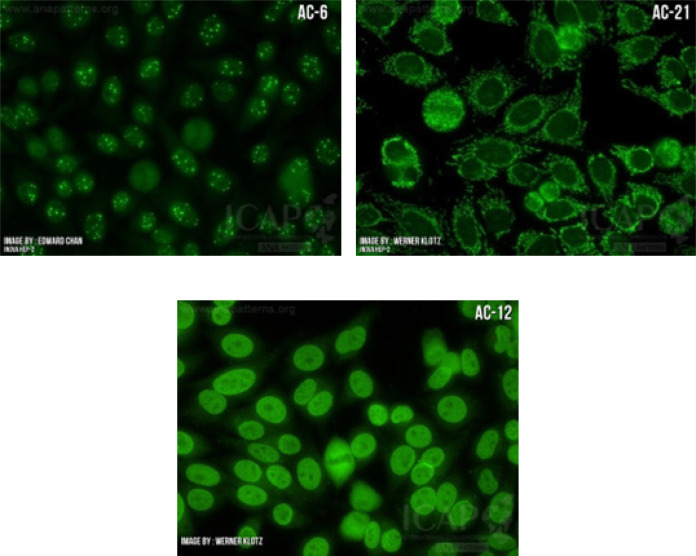
AC-12: anti-Gp 210 antibodies; nuclear envelope reveals punctate staining in interphase cells.^3^ AC-6: anti-Sp100 antibodies; countable (6 to 20) nuclear discrete dots. AC-21: anti-mitochondrial antibodies; coarse filamentous staining extending throughout the cytoplasm.

**Table 1.** Demographic and laboratory findings in patients with PBC and overlap PBC-AIH.

**Variables**	**Total patients (n = 19)**	**PBC patients (n = 14)**	**Patients with overlap PBC-AIH (n = 5)**
**Mean age (years)**	58,9	61,1	53
**Female, n (%)**	17 (89.4%)	13 (92.8%)	4 (80%)
**AMA+**	17 (89.4%)	12 (85.7%)	5 (100%)
**Anti-gp210+**	11 (57.8%)	7 (50%)	4 (80%)
**Anti-Sp100+**	4 (21.1%)	2 (14.3%)	2 (40%)
**F-actin**	0 (0%)	0 (0%)	2 (40%)
**AST /ALT**	9 (47.4%)	5 (35.7%)	4 (80%)
**ALP**	16 (84.2%)	10 (81.4%)	4 (80%)
**GGT**	15 (78.9%)	11 (78.5%)	4 (80%)
**TB**	10 (81.4%)	10 (71.4%)	3 (75%)

**Table 2.** PBC Patients are divided into groups depending on the positivity of antibodies.

**Sample (n=19)**	**AMA (n=17)**	**anti-gp210 (n=11)**	**anti-Sp100 (n=4)**
**G1 n=2 (10.5%)**	Negative n=2	Positive n=2 (18.2%)	Positive n=0
**G2 n=11 (57.9%)**	Positive n=11(64.7%)	Positive n=9 (81.8%)	Positive n=4 (100%)
**G3 n=5 (26.3%)**	Positive n=5 (29.4%)	Negative n=5	Negative n=5
**G4 n=1 (5.3%)**	Negative n=1	Negative n=1	Negative n=1
